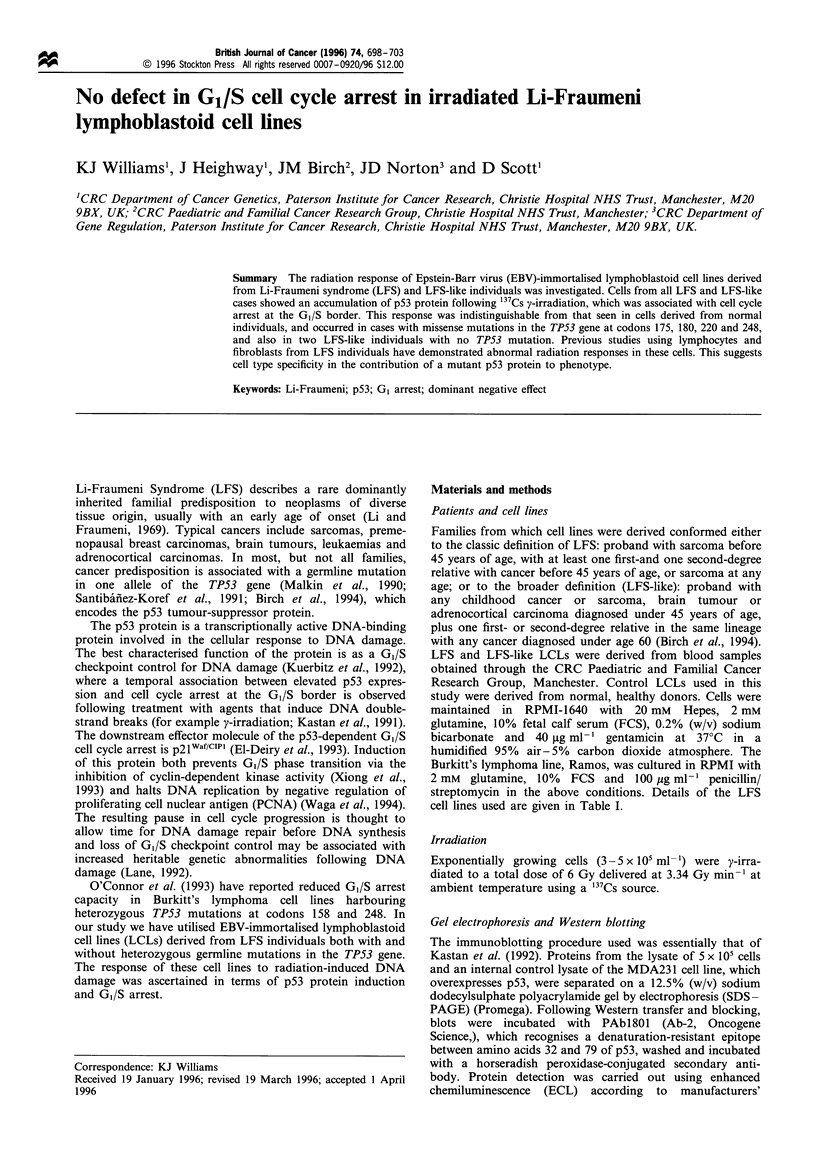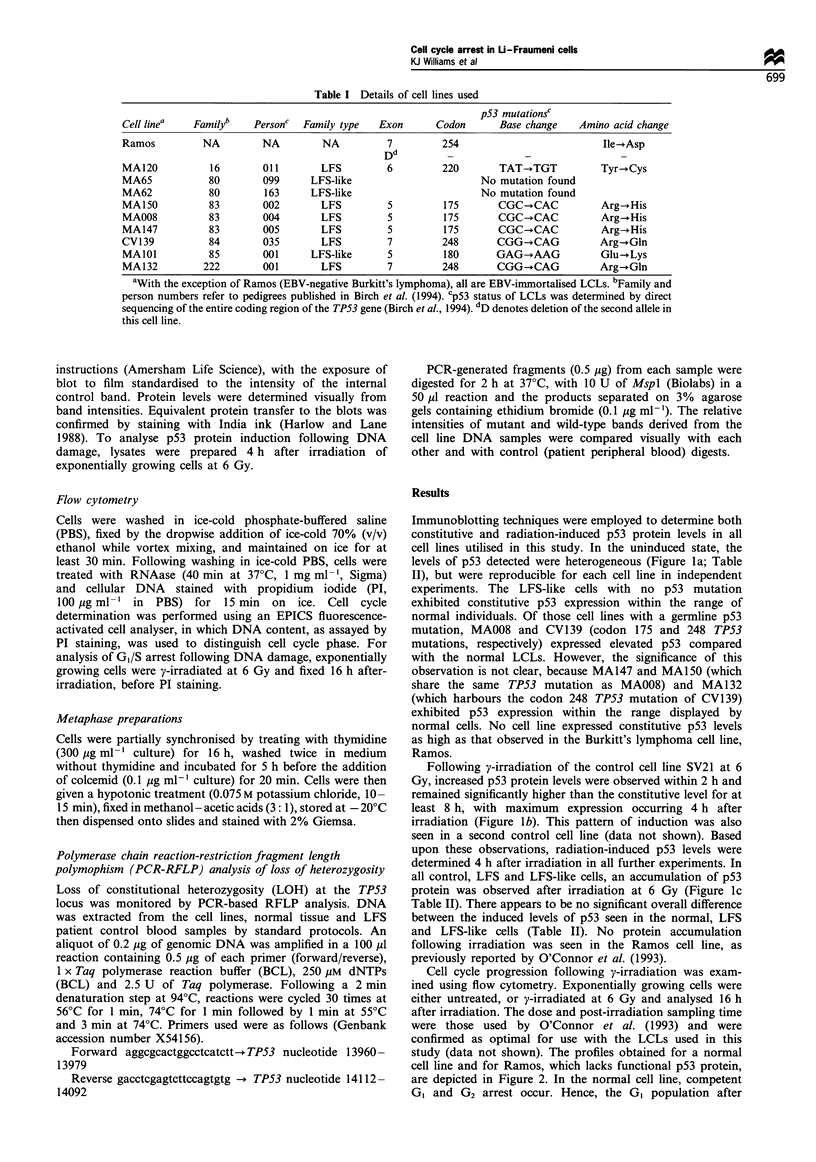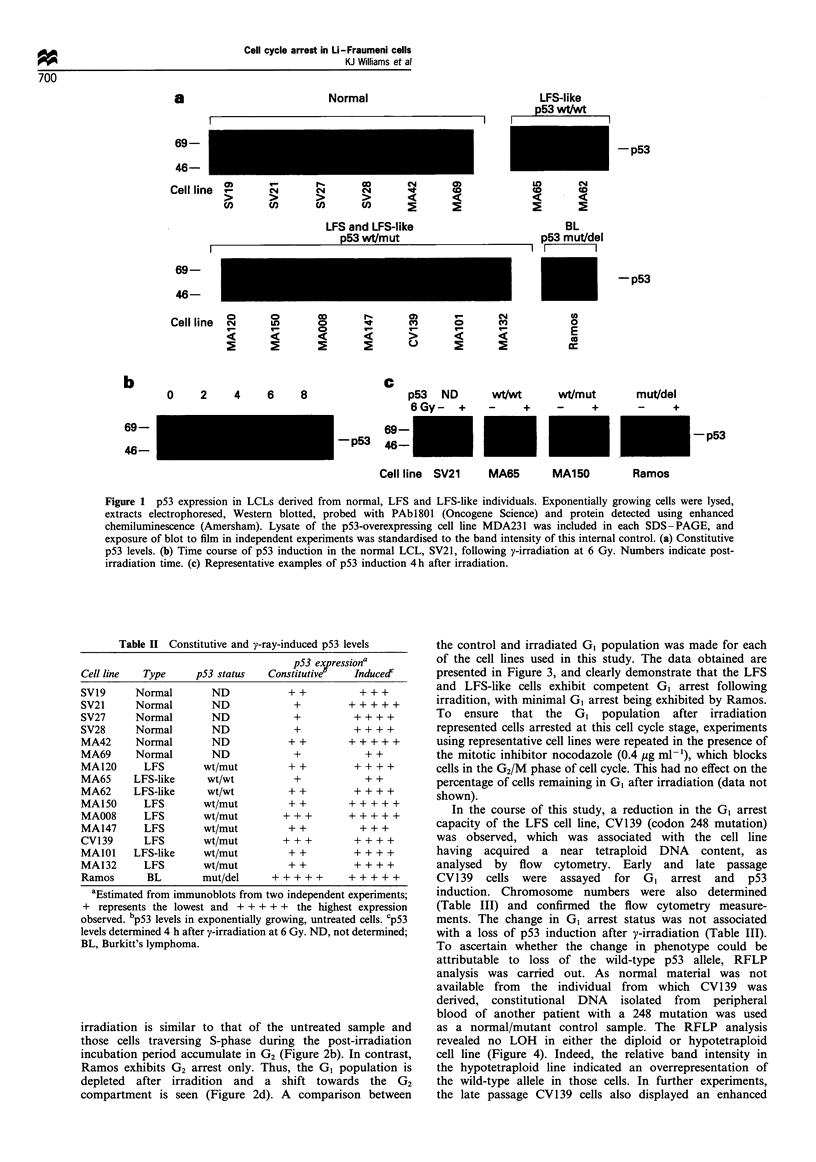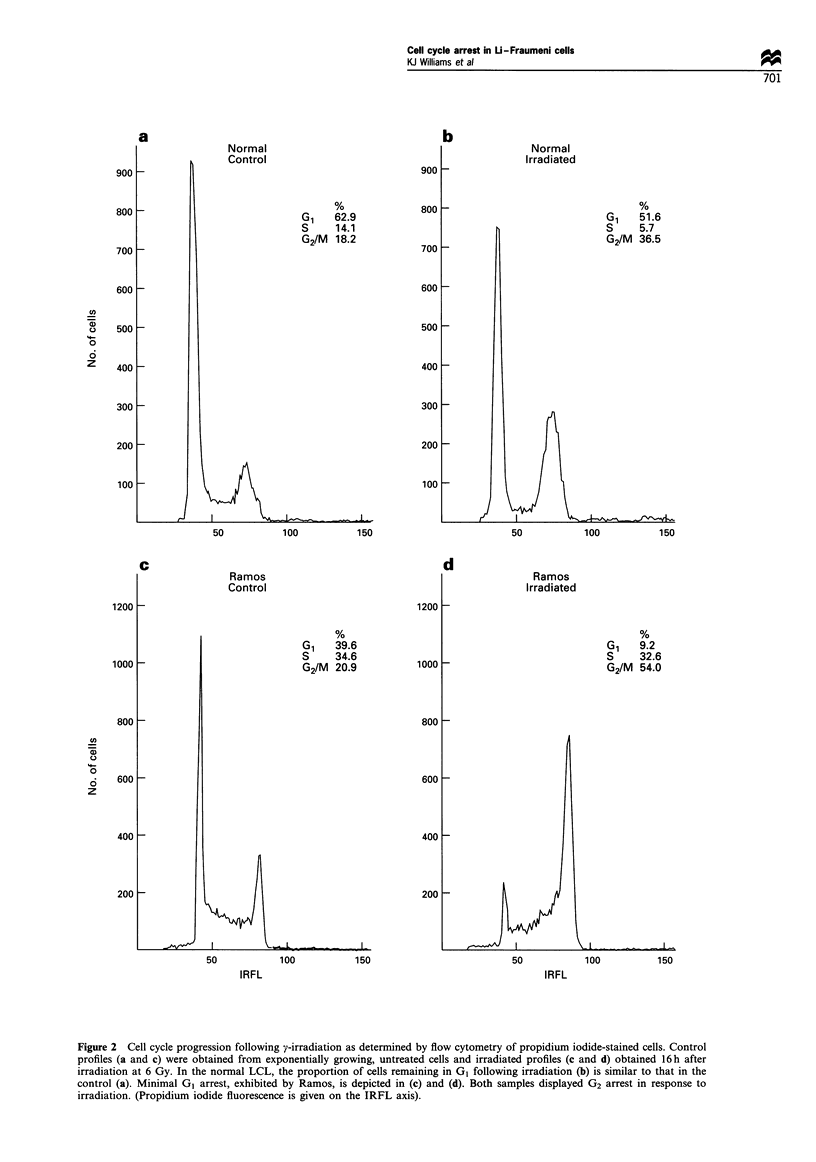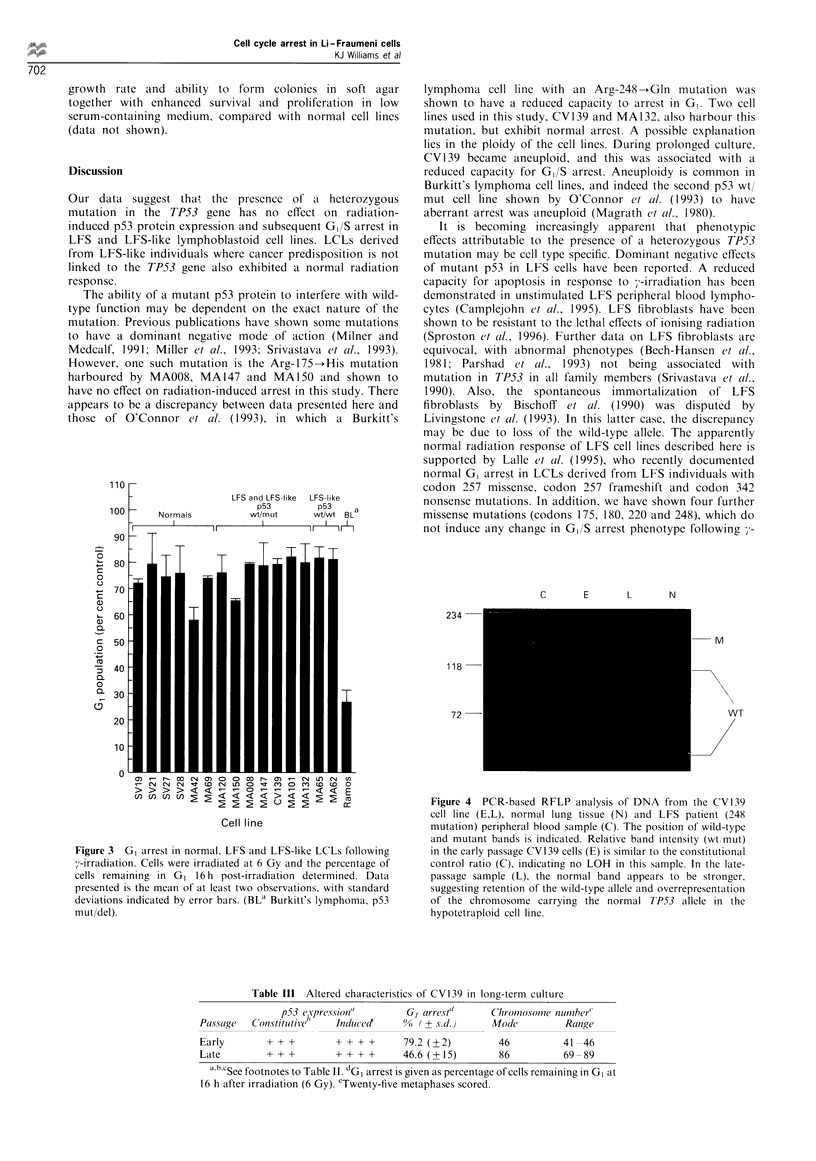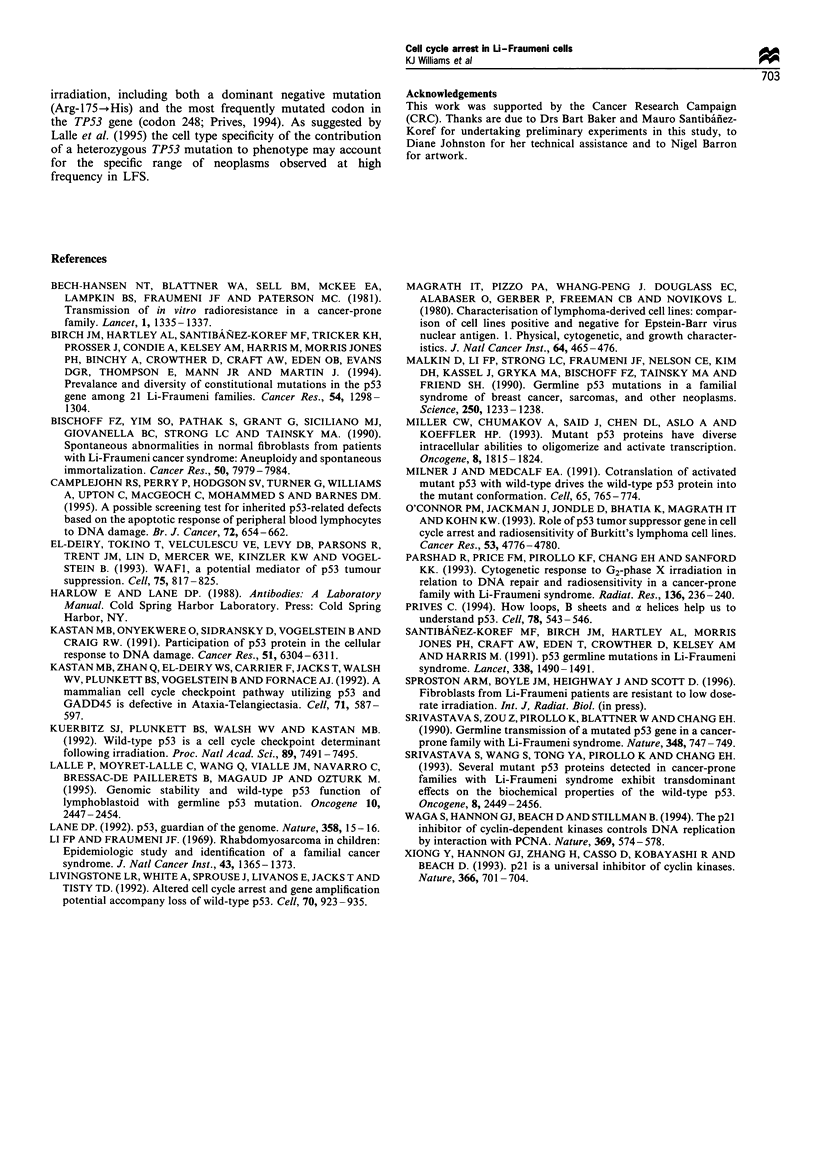# No defect in G1/S cell cycle arrest in irradiated Li-Fraumeni lymphoblastoid cell lines.

**DOI:** 10.1038/bjc.1996.424

**Published:** 1996-09

**Authors:** K. J. Williams, J. Heighway, J. M. Birch, J. D. Norton, D. Scott

**Affiliations:** CRC Department of Cancer Genetics, Paterson Institute for Cancer Research, Christie Hospital NHS Trust, Manchester, UK.

## Abstract

**Images:**


British Journal of Cancer (1996) 74, 698-703
© 1996 Stockton Press All rights reserved 0007-0920/96 $12.00

### No defect in $G_1$/S cell cycle arrest in irradiated Li-Fraumeni lymphoblastoid cell lines

KJ Williams[1], J Heighway[1], JM Birch[2], JD Norton[3] and D Scott[1]

*[1]CRC Department of Cancer Genetics, Paterson Institute for Cancer Research, Christie Hospital NHS Trust, Manchester, M20 9BX, UK; [2]CRC Paediatric and Familial Cancer Research Group, Christie Hospital NHS Trust, Manchester; [3]CRC Department of Gene Regulation, Paterson Institute for Cancer Research, Christie Hospital NHS Trust, Manchester, M20 9BX, UK.*

**Summary** The radiation response of Epstein-Barr virus (EBV)-immortalised lymphoblastoid cell lines derived from Li-Fraumeni syndrome (LFS) and LFS-like individuals was investigated. Cells from all LFS and LFS-like cases showed an accumulation of p53 protein following $^{137}$Cs $\gamma$-irradiation, which was associated with cell cycle arrest at the $G_1$/S border. This response was indistinguishable from that seen in cells derived from normal individuals, and occurred in cases with missense mutations in the *TP53* gene at codons 175, 180, 220 and 248, and also in two LFS-like individuals with no *TP53* mutation. Previous studies using lymphocytes and fibroblasts from LFS individuals have demonstrated abnormal radiation responses in these cells. This suggests cell type specificity in the contribution of a mutant p53 protein to phenotype.

**Keywords:** Li-Fraumeni; p53; $G_1$ arrest; dominant negative effect

Li-Fraumeni Syndrome (LFS) describes a rare dominantly inherited familial predisposition to neoplasms of diverse tissue origin, usually with an early age of onset (Li and Fraumeni, 1969). Typical cancers include sarcomas, premenopausal breast carcinomas, brain tumours, leukaemias and adrenocortical carcinomas. In most, but not all families, cancer predisposition is associated with a germline mutation in one allele of the *TP53* gene (Malkin *et al.*, 1990; Santibáñez-Koref *et al.*, 1991; Birch *et al.*, 1994), which encodes the p53 tumour-suppressor protein.

The p53 protein is a transcriptionally active DNA-binding protein involved in the cellular response to DNA damage. The best characterised function of the protein is as a $G_1$/S checkpoint control for DNA damage (Kuerbitz *et al.*, 1992), where a temporal association between elevated p53 expression and cell cycle arrest at the $G_1$/S border is observed following treatment with agents that induce DNA double-strand breaks (for example $\gamma$-irradiation; Kastan *et al.*, 1991). The downstream effector molecule of the p53-dependent $G_1$/S cell cycle arrest is $p21^{Waf/CIP1}$ (El-Deiry *et al.*, 1993). Induction of this protein both prevents $G_1$/S phase transition via the inhibition of cyclin-dependent kinase activity (Xiong *et al.*, 1993) and halts DNA replication by negative regulation of proliferating cell nuclear antigen (PCNA) (Waga *et al.*, 1994). The resulting pause in cell cycle progression is thought to allow time for DNA damage repair before DNA synthesis and loss of $G_1$/S checkpoint control may be associated with increased heritable genetic abnormalities following DNA damage (Lane, 1992).

O'Connor *et al.* (1993) have reported reduced $G_1$/S arrest capacity in Burkitt's lymphoma cell lines harbouring heterozygous *TP53* mutations at codons 158 and 248. In our study we have utilised EBV-immortalised lymphoblastoid cell lines (LCLs) derived from LFS individuals both with and without heterozygous germline mutations in the *TP53* gene. The response of these cell lines to radiation-induced DNA damage was ascertained in terms of p53 protein induction and $G_1$/S arrest.

Correspondence: KJ Williams
Received 19 January 1996; revised 19 March 1996; accepted 1 April 1996

### Materials and methods

### *Patients and cell lines*

Families from which cell lines were derived conformed either to the classic definition of LFS: proband with sarcoma before 45 years of age, with at least one first-and one second-degree relative with cancer before 45 years of age, or sarcoma at any age; or to the broader definition (LFS-like): proband with any childhood cancer or sarcoma, brain tumour or adrenocortical carcinoma diagnosed under 45 years of age, plus one first- or second-degree relative in the same lineage with any cancer diagnosed under age 60 (Birch *et al.*, 1994). LFS and LFS-like LCLs were derived from blood samples obtained through the CRC Paediatric and Familial Cancer Research Group, Manchester. Control LCLs used in this study were derived from normal, healthy donors. Cells were maintained in RPMI-1640 with 20 mM Hepes, 2 mM glutamine, 10% fetal calf serum (FCS), 0.2% (w/v) sodium bicarbonate and 40 µg ml$^{-1}$ gentamicin at 37°C in a humidified 95% air-5% carbon dioxide atmosphere. The Burkitt's lymphoma line, Ramos, was cultured in RPMI with 2 mM glutamine, 10% FCS and 100 µg ml$^{-1}$ penicillin/streptomycin in the above conditions. Details of the LFS cell lines used are given in Table I.

### *Irradiation*

Exponentially growing cells ($3-5\times10^5$ ml$^{-1}$) were $\gamma$-irradiated to a total dose of 6 Gy delivered at 3.34 Gy min$^{-1}$ at ambient temperature using a $^{137}$Cs source.

### *Gel electrophoresis and Western blotting*

The immunoblotting procedure used was essentially that of Kastan *et al.* (1992). Proteins from the lysate of $5\times10^5$ cells and an internal control lysate of the MDA231 cell line, which overexpresses p53, were separated on a 12.5% (w/v) sodium dodecylsulphate polyacrylamide gel by electrophoresis (SDS-PAGE) (Promega). Following Western transfer and blocking, blots were incubated with PAb1801 (Ab-2, Oncogene Science,), which recognises a denaturation-resistant epitope between amino acids 32 and 79 of p53, washed and incubated with a horseradish peroxidase-conjugated secondary antibody. Protein detection was carried out using enhanced chemiluminescence (ECL) according to manufacturers'

**Table I** Details of cell lines used

| Cell line[a] | Family[b] | Person[c] | Family type | Exon | Codon | p53 mutations[c] Base change | Amino acid change |
|---|---|---|---|---|---|---|---|
| Ramos | NA | NA | NA | 7 | 254 | | Ile→Asp |
| | | | | D[d] | – | – | – |
| MA120 | 16 | 011 | LFS | 6 | 220 | TAT→TGT | Tyr→Cys |
| MA65 | 80 | 099 | LFS-like | | | No mutation found | |
| MA62 | 80 | 163 | LFS-like | | | No mutation found | |
| MA150 | 83 | 002 | LFS | 5 | 175 | CGC→CAC | Arg→His |
| MA008 | 83 | 004 | LFS | 5 | 175 | CGC→CAC | Arg→His |
| MA147 | 83 | 005 | LFS | 5 | 175 | CGC→CAC | Arg→His |
| CV139 | 84 | 035 | LFS | 7 | 248 | CGG→CAG | Arg→Gln |
| MA101 | 85 | 001 | LFS-like | 5 | 180 | GAG→AAG | Glu→Lys |
| MA132 | 222 | 001 | LFS | 7 | 248 | CGG→CAG | Arg→Gln |

[a]With the exception of Ramos (EBV-negative Burkitt's lymphoma), all are EBV-immortalised LCLs. [b]Family and person numbers refer to pedigrees published in Birch *et al.* (1994). [c]p53 status of LCLs was determined by direct sequencing of the entire coding region of the *TP53* gene (Birch *et al.*, 1994). [d]D denotes deletion of the second allele in this cell line.

instructions (Amersham Life Science), with the exposure of blot to film standardised to the intensity of the internal control band. Protein levels were determined visually from band intensities. Equivalent protein transfer to the blots was confirmed by staining with India ink (Harlow and Lane 1988). To analyse p53 protein induction following DNA damage, lysates were prepared 4 h after irradiation of exponentially growing cells at 6 Gy.

*Flow cytometry*

Cells were washed in ice-cold phosphate-buffered saline (PBS), fixed by the dropwise addition of ice-cold 70% (v/v) ethanol while vortex mixing, and maintained on ice for at least 30 min. Following washing in ice-cold PBS, cells were treated with RNAase (40 min at 37°C, 1 mg ml$^{-1}$, Sigma) and cellular DNA stained with propidium iodide (PI, 100 $\mu$g ml$^{-1}$ in PBS) for 15 min on ice. Cell cycle determination was performed using an EPICS fluorescence-activated cell analyser, in which DNA content, as assayed by PI staining, was used to distinguish cell cycle phase. For analysis of $G_1$/S arrest following DNA damage, exponentially growing cells were $\gamma$-irradiated at 6 Gy and fixed 16 h after-irradiation, before PI staining.

*Metaphase preparations*

Cells were partially synchronised by treating with thymidine (300 $\mu$g ml$^{-1}$ culture) for 16 h, washed twice in medium without thymidine and incubated for 5 h before the addition of colcemid (0.1 $\mu$g ml$^{-1}$ culture) for 20 min. Cells were then given a hypotonic treatment (0.075 M potassium chloride, 10–15 min), fixed in methanol–acetic acids (3 : 1), stored at −20°C then dispensed onto slides and stained with 2% Giemsa.

*Polymerase chain reaction-restriction fragment length polymophism (PCR-RFLP) analysis of loss of heterozygosity*

Loss of constitutional heterozygosity (LOH) at the *TP53* locus was monitored by PCR-based RFLP analysis. DNA was extracted from the cell lines, normal tissue and LFS patient control blood samples by standard protocols. An aliquot of 0.2 $\mu$g of genomic DNA was amplified in a 100 $\mu$l reaction containing 0.5 $\mu$g of each primer (forward/reverse), 1 × *Taq* polymerase reaction buffer (BCL), 250 $\mu$M dNTPs (BCL) and 2.5 U of *Taq* polymerase. Following a 2 min denaturation step at 94°C, reactions were cycled 30 times at 56°C for 1 min, 74°C for 1 min followed by 1 min at 55°C and 3 min at 74°C. Primers used were as follows (Genbank accession number X54156).

Forward aggcgcactggcctcatctt→*TP53* nucleotide 13960–13979

Reverse gacctcgagtcttccagtgtg → *TP53* nucleotide 14112–14092

PCR-generated fragments (0.5 $\mu$g) from each sample were digested for 2 h at 37°C, with 10 U of *MspI* (Biolabs) in a 50 $\mu$l reaction and the products separated on 3% agarose gels containing ethidium bromide (0.1 $\mu$g ml$^{-1}$). The relative intensities of mutant and wild-type bands derived from the cell line DNA samples were compared visually with each other and with control (patient peripheral blood) digests.

### Results

Immunoblotting techniques were employed to determine both constitutive and radiation-induced p53 protein levels in all cell lines utilised in this study. In the uninduced state, the levels of p53 detected were heterogeneous (Figure 1a; Table II), but were reproducible for each cell line in independent experiments. The LFS-like cells with no p53 mutation exhibited constitutive p53 expression within the range of normal individuals. Of those cell lines with a germline p53 mutation, MA008 and CV139 (codon 175 and 248 *TP53* mutations, respectively) expressed elevated p53 compared with the normal LCLs. However, the significance of this observation is not clear, because MA147 and MA150 (which share the same *TP53* mutation as MA008) and MA132 (which harbours the codon 248 *TP53* mutation of CV139) exhibited p53 expression within the range displayed by normal cells. No cell line expressed constitutive p53 levels as high as that observed in the Burkitt's lymphoma cell line, Ramos.

Following $\gamma$-irradiation of the control cell line SV21 at 6 Gy, increased p53 protein levels were observed within 2 h and remained significantly higher than the constitutive level for at least 8 h, with maximum expression occurring 4 h after irradiation (Figure 1*b*). This pattern of induction was also seen in a second control cell line (data not shown). Based upon these observations, radiation-induced p53 levels were determined 4 h after irradiation in all further experiments. In all control, LFS and LFS-like cells, an accumulation of p53 protein was observed after irradiation at 6 Gy (Figure 1c Table II). There appears to be no significant overall difference between the induced levels of p53 seen in the normal, LFS and LFS-like cells (Table II). No protein accumulation following irradiation was seen in the Ramos cell line, as previously reported by O'Connor *et al.* (1993).

Cell cycle progression following $\gamma$-irradiation was examined using flow cytometry. Exponentially growing cells were either untreated, or $\gamma$-irradiated at 6 Gy and analysed 16 h after irradiation. The dose and post-irradiation sampling time were those used by O'Connor *et al.* (1993) and were confirmed as optimal for use with the LCLs used in this study (data not shown). The profiles obtained for a normal cell line and for Ramos, which lacks functional p53 protein, are depicted in Figure 2. In the normal cell line, competent $G_1$ and $G_2$ arrest occur. Hence, the $G_1$ population after

**Figure 1** p53 expression in LCLs derived from normal, LFS and LFS-like individuals. Exponentially growing cells were lysed, extracts electrophoresed, Western blotted, probed with PAb1801 (Oncogene Science) and protein detected using enhanced chemiluminescence (Amersham). Lysate of the p53-overexpressing cell line MDA231 was included in each SDS–PAGE, and exposure of blot to film in independent experiments was standardised to the band intensity of this internal control. (**a**) Constitutive p53 levels. (**b**) Time course of p53 induction in the normal LCL, SV21, following $\gamma$-irradiation at 6 Gy. Numbers indicate post-irradiation time. (**c**) Representative examples of p53 induction 4 h after irradiation.

**Table II** Constitutive and $\gamma$-ray-induced p53 levels

| *Cell line* | *Type* | *p53 status* | *p53 expression*[a] *Constitutive*[b] | *Induced*[c] |
|---|---|---|---|---|
| SV19 | Normal | ND | + + | + + + |
| SV21 | Normal | ND | + | + + + + + |
| SV27 | Normal | ND | + | + + + + |
| SV28 | Normal | ND | + | + + + + |
| MA42 | Normal | ND | + + | + + + + + |
| MA69 | Normal | ND | + | + + |
| MA120 | LFS | wt/mut | + + | + + + + |
| MA65 | LFS-like | wt/wt | + | + + |
| MA62 | LFS-like | wt/wt | + + | + + + + |
| MA150 | LFS | wt/mut | + + | + + + + + |
| MA008 | LFS | wt/mut | + + + | + + + + + |
| MA147 | LFS | wt/mut | + + | + + + |
| CV139 | LFS | wt/mut | + + + | + + + + |
| MA101 | LFS-like | wt/mut | + + | + + + + |
| MA132 | LFS | wt/mut | + + | + + + + |
| Ramos | BL | mut/del | + + + + + | + + + + + |

[a]Estimated from immunoblots from two independent experiments; + represents the lowest and + + + + + the highest expression observed. [b]p53 levels in exponentially growing, untreated cells. [c]p53 levels determined 4 h after $\gamma$-irradiation at 6 Gy. ND, not determined; BL, Burkitt's lymphoma.

irradiation is similar to that of the untreated sample and those cells traversing S-phase during the post-irradiation incubation period accumulate in $G_2$ (Figure 2b). In contrast, Ramos exhibits $G_2$ arrest only. Thus, the $G_1$ population is depleted after irradition and a shift towards the $G_2$ compartment is seen (Figure 2d). A comparison between the control and irradiated $G_1$ population was made for each of the cell lines used in this study. The data obtained are presented in Figure 3, and clearly demonstrate that the LFS and LFS-like cells exhibit competent $G_1$ arrest following irradition, with minimal $G_1$ arrest being exhibited by Ramos. To ensure that the $G_1$ population after irradiation represented cells arrested at this cell cycle stage, experiments using representative cell lines were repeated in the presence of the mitotic inhibitor nocodazole (0.4 $\mu g\ ml^{-1}$), which blocks cells in the $G_2$/M phase of cell cycle. This had no effect on the percentage of cells remaining in $G_1$ after irradiation (data not shown).

In the course of this study, a reduction in the $G_1$ arrest capacity of the LFS cell line, CV139 (codon 248 mutation) was observed, which was associated with the cell line having acquired a near tetraploid DNA content, as analysed by flow cytometry. Early and late passage CV139 cells were assayed for $G_1$ arrest and p53 induction. Chromosome numbers were also determined (Table III) and confirmed the flow cytometry measurements. The change in $G_1$ arrest status was not associated with a loss of p53 induction after $\gamma$-irradiation (Table III). To ascertain whether the change in phenotype could be attributable to loss of the wild-type p53 allele, RFLP analysis was carried out. As normal material was not available from the individual from which CV139 was derived, constitutional DNA isolated from peripheral blood of another patient with a 248 mutation was used as a normal/mutant control sample. The RFLP analysis revealed no LOH in either the diploid or hypotetraploid cell line (Figure 4). Indeed, the relative band intensity in the hypotetraploid line indicated an overrepresentation of the wild-type allele in those cells. In further experiments, the late passage CV139 cells also displayed an enhanced

**Figure 2** Cell cycle progression following $\gamma$-irradiation as determined by flow cytometry of propidium iodide-stained cells. Control profiles (**a** and **c**) were obtained from exponentially growing, untreated cells and irradiated profiles (**c** and **d**) obtained 16 h after irradiation at 6 Gy. In the normal LCL, the proportion of cells remaining in $G_1$ following irradiation (**b**) is similar to that in the control (**a**). Minimal $G_1$ arrest, exhibited by Ramos, is depicted in (**c**) and (**d**). Both samples displayed $G_2$ arrest in response to irradiation. (Propidium iodide fluorescence is given on the IRFL axis).

growth rate and ability to form colonies in soft agar together with enhanced survival and proliferation in low serum-containing medium, compared with normal cell lines (data not shown).

### Discussion

Our data suggest that the presence of a heterozygous mutation in the *TP53* gene has no effect on radiation-induced p53 protein expression and subsequent $G_1/S$ arrest in LFS and LFS-like lymphoblastoid cell lines. LCLs derived from LFS-like individuals where cancer predisposition is not linked to the *TP53* gene also exhibited a normal radiation response.

The ability of a mutant p53 protein to interfere with wild-type function may be dependent on the exact nature of the mutation. Previous publications have shown some mutations to have a dominant negative mode of action (Milner and Medcalf, 1991; Miller *et al*., 1993; Srivastava *et al*., 1993). However, one such mutation is the Arg-175→His mutation harboured by MA008, MA147 and MA150 and shown to have no effect on radiation-induced arrest in this study. There appears to be a discrepancy between data presented here and those of O'Connor *et al*. (1993), in which a Burkitt's lymphoma cell line with an Arg-248→Gln mutation was shown to have a reduced capacity to arrest in $G_1$. Two cell lines used in this study, CV139 and MA132, also harbour this mutation, but exhibit normal arrest. A possible explanation lies in the ploidy of the cell lines. During prolonged culture, CV139 became aneuploid, and this was associated with a reduced capacity for $G_1/S$ arrest. Aneuploidy is common in Burkitt's lymphoma cell lines, and indeed the second p53 wt/mut cell line shown by O'Connor *et al*. (1993) to have aberrant arrest was aneuploid (Magrath *et al*., 1980).

It is becoming increasingly apparent that phenotypic effects attributable to the presence of a heterozygous *TP53* mutation may be cell type specific. Dominant negative effects of mutant p53 in LFS cells have been reported. A reduced capacity for apoptosis in response to $\gamma$-irradiation has been demonstrated in unstimulated LFS peripheral blood lymphocytes (Camplejohn *et al*., 1995). LFS fibroblasts have been shown to be resistant to the lethal effects of ionising radiation (Sproston *et al*., 1996). Further data on LFS fibroblasts are equivocal, with abnormal phenotypes (Bech-Hansen *et al*., 1981; Parshad *et al*., 1993) not being associated with mutation in *TP53* in all family members (Srivastava *et al*., 1990). Also, the spontaneous immortalization of LFS fibroblasts by Bischoff *et al*. (1990) was disputed by Livingstone *et al*. (1993). In this latter case, the discrepancy may be due to loss of the wild-type allele. The apparently normal radiation response of LFS cell lines described here is supported by Lalle *et al*. (1995), who recently documented normal $G_1$ arrest in LCLs derived from LFS individuals with codon 257 missense, codon 257 frameshift and codon 342 nonsense mutations. In addition, we have shown four further missense mutations (codons 175, 180, 220 and 248), which do not induce any change in $G_1/S$ arrest phenotype following $\gamma$-

**Figure 3** $G_1$ arrest in normal, LFS and LFS-like LCLs following $\gamma$-irradiation. Cells were irradiated at 6 Gy and the percentage of cells remaining in $G_1$ 16 h post-irradiation determined. Data presented is the mean of at least two observations, with standard deviations indicated by error bars. (BL[a] Burkitt's lymphoma, p53 mut/del).

**Figure 4** PCR-based RFLP analysis of DNA from the CV139 cell line (E,L), normal lung tissue (N) and LFS patient (248 mutation) peripheral blood sample (C). The position of wild-type and mutant bands is indicated. Relative band intensity (wt/mut) in the early passage CV139 cells (E) is similar to the constitutional control ratio (C), indicating no LOH in this sample. In the late-passage sample (L), the normal band appears to be stronger, suggesting retention of the wild-type allele and overrepresentation of the chromosome carrying the normal *TP53* allele in the hypotetraploid cell line.

**Table III** Altered characteristics of CV139 in long-term culture

| *Passage* | *p53 expression*[a] *Constitutive*[b] | *Induced*[c] | $G_1$ *arrest*[d] % ( ± *s.d.*) | *Chromosome number*[e] *Mode* | *Range* |
|---|---|---|---|---|---|
| Early | + + + | + + + + | 79.2 (±2) | 46 | 41–46 |
| Late | + + + | + + + + | 46.6 (±15) | 86 | 69–89 |

[a,b,c]See footnotes to Table II. [d]$G_1$ arrest is given as percentage of cells remaining in $G_1$ at 16 h after irradiation (6 Gy). [e]Twenty-five metaphases scored.

irradiation, including both a dominant negative mutation (Arg-175→His) and the most frequently mutated codon in the *TP53* gene (codon 248; Prives, 1994). As suggested by Lalle *et al.* (1995) the cell type specificity of the contribution of a heterozygous *TP53* mutation to phenotype may account for the specific range of neoplasms observed at high frequency in LFS.

**Acknowledgements**

This work was supported by the Cancer Research Campaign (CRC). Thanks are due to Drs Bart Baker and Mauro Santibáñez-Koref for undertaking preliminary experiments in this study, to Diane Johnston for her technical assistance and to Nigel Barron for artwork.

### References

BECH-HANSEN NT, BLATTNER WA, SELL BM, MCKEE EA, LAMPKIN BS, FRAUMENI JF AND PATERSON MC. (1981). Transmission of *in vitro* radioresistance in a cancer-prone family. *Lancet*, **1**, 1335–1337.

BIRCH JM, HARTLEY AL, SANTIBÁÑEZ-KOREF MF, TRICKER KH, PROSSER J, CONDIE A, KELSEY AM, HARRIS M, MORRIS JONES PH, BINCHY A, CROWTHER D, CRAFT AW, EDEN OB, EVANS DGR, THOMPSON E, MANN JR AND MARTIN J. (1994). Prevalance and diversity of constitutional mutations in the p53 gene among 21 Li-Fraumeni families. *Cancer Res.*, **54**, 1298–1304.

BISCHOFF FZ, YIM SO, PATHAK S, GRANT G, SICILIANO MJ, GIOVANELLA BC, STRONG LC AND TAINSKY MA. (1990). Spontaneous abnormalities in normal fibroblasts from patients with Li-Fraumeni cancer syndrome: Aneuploidy and spontaneous immortalization. *Cancer Res.*, **50**, 7979–7984.

CAMPLEJOHN RS, PERRY P, HODGSON SV, TURNER G, WILLIAMS A, UPTON C, MACGEOCH C, MOHAMMED S AND BARNES DM. (1995). A possible screening test for inherited p53-related defects based on the apoptotic response of peripheral blood lymphocytes to DNA damage. *Br. J. Cancer*, **72**, 654–662.

EL-DEIRY, TOKINO T, VELCULESCU VE, LEVY DB, PARSONS R, TRENT JM, LIN D, MERCER WE, KINZLER KW AND VOGELSTEIN B. (1993). WAF1, a potential mediator of p53 tumour suppression. *Cell*, **75**, 817–825.

HARLOW E AND LANE DP. (1988). *Antibodies: A Laboratory Manual.* Cold Spring Harbor Laboratory. Press: Cold Spring Harbor, NY.

KASTAN MB, ONYEKWERE O, SIDRANSKY D, VOGELSTEIN B AND CRAIG RW. (1991). Participation of p53 protein in the cellular response to DNA damage. *Cancer Res.*, **51**, 6304–6311.

KASTAN MB, ZHAN Q, EL-DEIRY WS, CARRIER F, JACKS T, WALSH WV, PLUNKETT BS, VOGELSTEIN B AND FORNACE AJ. (1992). A mammalian cell cycle checkpoint pathway utilizing p53 and GADD45 is defective in Ataxia-Telangiectasia. *Cell*, **71**, 587–597.

KUERBITZ SJ, PLUNKETT BS, WALSH WV AND KASTAN MB. (1992). Wild-type p53 is a cell cycle checkpoint determinant following irradiation. *Proc. Natl Acad. Sci.*, **89**, 7491–7495.

LALLE P, MOYRET-LALLE C, WANG Q, VIALLE JM, NAVARRO C, BRESSAC-DE PAILLERETS B, MAGAUD JP AND OZTURK M. (1995). Genomic stability and wild-type p53 function of lymphoblastoid with germline p53 mutation. *Oncogene* **10**, 2447–2454.

LANE DP. (1992). p53, guardian of the genome. *Nature*, **358**, 15–16.

LI FP AND FRAUMENI JF. (1969). Rhabdomyosarcoma in children: Epidemiologic study and identification of a familial cancer syndrome. *J. Natl Cancer Inst.*, **43**, 1365–1373.

LIVINGSTONE LR, WHITE A, SPROUSE J, LIVANOS E, JACKS T AND TISTY TD. (1992). Altered cell cycle arrest and gene amplification potential accompany loss of wild-type p53. *Cell*, **70**, 923–935.

MAGRATH IT, PIZZO PA, WHANG-PENG J. DOUGLASS EC, ALABASER O, GERBER P, FREEMAN CB AND NOVIKOVS L. (1980). Characterisation of lymphoma-derived cell lines: comparison of cell lines positive and negative for Epstein-Barr virus nuclear antigen. 1. Physical, cytogenetic, and growth characteristics. *J. Natl Cancer Inst.*, **64**, 465–476.

MALKIN D, LI FP, STRONG LC, FRAUMENI JF, NELSON CE, KIM DH, KASSEL J, GRYKA MA, BISCHOFF FZ, TAINSKY MA AND FRIEND SH. (1990). Germline p53 mutations in a familial syndrome of breast cancer, sarcomas, and other neoplasms. *Science*, **250**, 1233–1238.

MILLER CW, CHUMAKOV A, SAID J, CHEN DL, ASLO A AND KOEFFLER HP. (1993). Mutant p53 proteins have diverse intracellular abilities to oligomerize and activate transcription. *Oncogene*, **8**, 1815–1824.

MILNER J AND MEDCALF EA. (1991). Cotranslation of activated mutant p53 with wild-type drives the wild-type p53 protein into the mutant conformation. *Cell*, 65, 765–774.

O'CONNOR PM, JACKMAN J, JONDLE D, BHATIA K, MAGRATH IT AND KOHN KW. (1993). Role of p53 tumor suppressor gene in cell cycle arrest and radiosensitivity of Burkitt's lymphoma cell lines. *Cancer Res.*, **53**, 4776–4780.

PARSHAD R, PRICE FM, PIROLLO KF, CHANG EH AND SANFORD KK. (1993). Cytogenetic response to $G_2$-phase X irradiation in relation to DNA repair and radiosensitivity in a cancer-prone family with Li-Fraumeni syndrome. *Radiat. Res.*, **136**, 236–240.

PRIVES C. (1994). How loops, B sheets and α helices help us to understand p53. *Cell*, **78**, 543–546.

SANTIBÁÑEZ-KOREF MF, BIRCH JM, HARTLEY AL, MORRIS JONES PH, CRAFT AW, EDEN T, CROWTHER D, KELSEY AM AND HARRIS M. (1991). p53 germline mutations in Li-Fraumeni syndrome. *Lancet*, **338**, 1490–1491.

SPROSTON ARM, BOYLE JM, HEIGHWAY J AND SCOTT D. (1996). Fibroblasts from Li-Fraumeni patients are resistant to low dose-rate irradiation. *Int. J, Radiat. Biol.* (in press).

SRIVASTAVA S, ZOU Z, PIROLLO K, BLATTNER W AND CHANG EH. (1990). Germline transmission of a mutated p53 gene in a cancer-prone family with Li-Fraumeni syndrome. *Nature*, **348**, 747–749.

SRIVASTAVA S, WANG S, TONG YA, PIROLLO K AND CHANG EH. (1993). Several mutant p53 proteins detected in cancer-prone families with Li-Fraumeni syndrome exhibit transdominant effects on the biochemical properties of the wild-type p53. *Oncogene*, **8**, 2449–2456.

WAGA S, HANNON GJ, BEACH D AND STILLMAN B. (1994). The p21 inhibitor of cyclin-dependent kinases controls DNA replication by interaction with PCNA. *Nature*, **369**, 574–578.

XIONG Y, HANNON GJ, ZHANG H, CASSO D, KOBAYASHI R AND BEACH D. (1993). p21 is a universal inhibitor of cyclin kinases. *Nature*, **366**, 701–704.